# National Trend and Characteristics of Acute Hepatitis C among HIV-Infected Individuals: A Matched Case-Control Study—Taiwan, 2001–2014

**DOI:** 10.1371/journal.pone.0139687

**Published:** 2015-10-06

**Authors:** Yi-Chun Lo, Mao-Song Tsai, Hsin-Yun Sun, Chien-Ching Hung, Jen-Hsiang Chuang

**Affiliations:** 1 Taiwan Centers for Disease Control, Taipei, Taiwan; 2 Department of Internal Medicine, National Taiwan University Hospital and National Taiwan University College of Medicine, Taipei, Taiwan; 3 Department of Internal Medicine, Far Eastern Memorial Hospital, New Taipei City, Taiwan; Fudan University, CHINA

## Abstract

**Background:**

Hepatitis C virus (HCV) infection has been increasingly recognized among HIV-infected men who have sex with men (MSM) worldwide. We investigated the trend of and factors associated with acute hepatitis C (AHC) among HIV-infected individuals in Taiwan.

**Methods:**

The National Disease Surveillance System collects characteristics of AHC, HIV, syphilis, and gonorrhea cases through mandatory reports and patient interviews. Reported AHC patients in 2014 were interviewed additionally on sexual and parenteral exposures. Information on HCV genotypes were collected from the largest medical center serving HIV-infected Taiwanese. We defined an HIV/AHC case as a documented negative HCV antibody test result followed within 12 months by a positive test in a previously reported HIV-infected individual. Each case was matched to two HIV-infected, non-AHC controls for age, age of HIV diagnosis, sex, transmission route, HIV diagnosis date, and county/city. Conditional logistic regression was used to identify associated characteristics.

**Results:**

During 2001–2014, 93 of 6,624 AHC reports were HIV/AHC cases; the annual case count increased from one in 2009 to 34 in 2014. All were males (81 [87%] MSM) aged 21–49 years with AHC diagnosed 2–5,923 days after HIV diagnoses. Sixty-eight (73%) lived in the Taipei metropolitan area. Detected HCV genotypes were 2a (n = 6), 1b (n = 5), 1b + 2a (n = 1) and 2b (n = 1). Among 28 HIV/AHC patients interviewed in 2014, 13 (46%) reported engaging in unprotected sex ≤3 months before AHC diagnosis. Seventy-nine HIV/AHC cases were matched to 158 controls. HIV/AHC was associated with recent syphilis (adjusted odds ratio [aOR], 10.9; 95% confidence interval [CI], 4.2–28.6) and last syphilis >6 months (aOR, 2.9; 95% CI, 1.2–6.9).

**Conclusions:**

HIV/AHC cases continued to increase particularly among sexually active HIV-infected MSM with a syphilis diagnosis in northern Taiwan. We recommend surveillance of associated behavioral and virologic characteristics and HCV counseling and testing for HIV-infected men in Taiwan.

## Introduction

Hepatitis C virus (HCV) infects an estimated 2.6%−3.1% of the world population and is a leading cause of liver disease [1].Without treatment, approximately 80% of patients with acute HCV infection progress to develop chronic infection and are at long-term risk for cirrhosis, hepatocellular carcinoma, and decompensated liver disease [2]. In HIV-infected individuals, HCV coinfection is common and the seroprevalence ranges from <5% in low-risk patients, 5−10% among MSM, to 50−90% among injection drug users (IDU) [3]. In HIV/HCV-coinfected individuals, HCV disease progresses more rapidly than HCV-monoinfected individuals and has become a leading cause of non-AIDS death [4–11].

Although HCV is primarily transmitted parenterally and sexual transmission of HCV in heterosexuals is considered rare [12], outbreaks of acute hepatitis C (AHC) through sexual transmission have been increasingly recognized among HIV-infected and HIV-uninfected men who have sex with men (MSM) in Europe, North America, and Australia [13–28]. Factors associated with sexually-transmitted AHC included unprotected receptive anal sex, use of sex toys, mucosally-administered recreational drug use (particularly methamphetamine use during sex), and concurrent or recent mucosally ulcerative sexually transmitted diseases (STDs) [29]. HIV-infected MSM carry approximately 4 times the risk of acute HCV acquisition than HIV-uninfected MSM, likely attributed to increased HCV susceptibility from HIV-induced compromise of humoral and cellular immunity [30–32].

In Asia, epidemiology of AHC among HIV-infected individuals has only been recently described in two hospital-based cohorts, one in Taiwan and the other in Japan [33–35]. Both studies demonstrated an increasing incidence of HCV seroconversion or AHC among HIV-infected MSM [33–34]. In the Taiwanese cohort, HCV seroconversion within 3 years was identified in 30 HIV-infected individuals (93.3% MSM) during 2001−2010 and was associated with recent syphilis acquisition [33]. In the Japanese cohort, AHC was diagnosed in 35 HIV-infected individuals during 2001−2012 who were predominantly MSM (96.6%) and antiretroviral therapy (ART)-experienced (90.6%) with well-suppressed plasma HIV RNA load (PVL) [35]. However, these studies are limited by the single-center design and the findings might not be generalized to HIV-infected individuals nationwide. We aimed to use the national surveillance data to investigate the trend of and factors associated with AHC among HIV-infected individuals in Taiwan.

## Materials and Methods

### Surveillance of AHC

Since 1999, the Communicable Disease Control Act in Taiwan has mandated healthcare providers to notify local public health departments of AHC cases within 7 days of diagnoses based on clinical assessment and commercially available HCV screening tests (generally enzyme immunoassays [EIA]). Healthcare providers are required to report demographic, clinical, and laboratory characteristics of AHC cases through the web-based, Taiwan Centers for Disease Control (TCDC)-operated Notifiable Disease Surveillance Systems (NDSS).

As a guidance to healthcare providers, TCDC has offered clinical and laboratory criteria for AHC reporting. During 1999−2014, the clinical criteria included the following conditions in an individual with a positive HCV antibody test result: (1) clinical presentations consistent with acute hepatitis; (2) a serum alanine aminotransferase (ALT) level ≥100 IU/L (included since July 1, 2006); (3) exclusion of chronic HCV infection and non-HCV causes of acute hepatitis; and (4) jaundice (included during November 2010−March 2014). The laboratory criteria included the following conditions: (1) documented HCV seroconversion, defined as a documented negative HCV antibody test result followed within 12 months by a positive test; and (2) a positive nucleic acid test for HCV RNA with a negative HCV antibody (included after March 6, 2014).

TCDC recommends reporting of AHC if a case meets all of the clinical criteria or any of the laboratory criteria. However, in practice the NDSS has accepted all AHC reports from healthcare providers even if the criteria are not fully met. The NDSS has also accepted cases with a positive HCV antibody test result and ALT level ≥100 IU/L with or without documented HCV seroconversion from blood donation centers. Notably, because excessive reporting of HCV EIA-positive cases from blood donation centers in 2013 (341 cases in 2013 compared with 32−83 cases annually during 2009−2011) raised concerns about false-positive results which was considered more often in blood donors [36], TCDC has recommended blood donation centers conduct recombinant immunoblot assay (RIBA) in HCV EIA-positive cases before reporting since 2014.

The NDSS further classified reported cases based on the TCDC’s AHC case definitions which had undergone 7 revisions during 1999−2014. Varied AHC case definitions have been used in published studies, such as HCV seroconversion ≤6 months or ≤12 months, or ALT level >100 IU/L followed by HCV seroconversion [33, 35, 37]. Because the NDSS readily collected data on HCV seroconversion within 12 months, for this study, we chose to ignore the NDSS case classification but follow the AHC case definitions established by the European AIDS Treatment Network [38]. Accordingly, we defined an HIV/AHC case as a documented negative HCV antibody test result followed within 12 months by a positive test (documented HCV seroconversion) in a previously reported HIV-infected individual. Because information on documented HCV seroconversion had not been collected until June 2001, only AHC cases in the NDSS reported during June 2001−December 2014 were analyzed to identify HIV/AHC cases.

The NDSS started to collect data on qualitative HCV RNA test results (positive, negative, or not conducted) among AHC cases from reporting healthcare facilities after March 6, 2014. Because the NDSS did not require physicians to report HCV viral load and genotypes among AHC cases, we collaborated with the National Taiwan University Hospital (NTUH), the largest medical center serving HIV-infected patients in the Taipei metropolitan area, to retrospectively collect data on HCV viral load and genotypes among HIV/AHC patients who received medical care at NTUH.

### Survey of sexual and parenteral exposures before AHC diagnosis

During March 7−December 31, 2014, a standardized questionnaire survey was administered by local health department staff through telephone interviews in all AHC cases with documented HCV seroconversion. Patients were asked about engagement in unprotected heterosexual or same-sex sex and the number of sex partners ≤3 months before AHC diagnosis, and parenteral exposures ≤6 months before AHC diagnosis.

### Surveillance of HIV infection, syphilis, and gonorrhea

In Taiwan, TCDC has mandated medical professionals to notify local public health departments of cases of HIV infection within 24 hours of diagnoses since 1985, and of syphilis and gonorrhea within 7 days of diagnoses since 1999. Surveillance of these diseases through the NDSS has been described in details [39]. In all reported HIV cases, self-reported information on sexual behaviors, injection drug use, and other risk factors is collected through face-to-face public health interviews. The NDSS also collected the patients’ CD4 count, PVL, and ART use every 3–6 months.

### Database linkage

To identify HIV/AHC cases and reports of syphilis and gonorrhea before AHC diagnoses, the NDSS databases of AHC (June 2001–2014), HIV infection (1985–2014), syphilis (1999–2014), and gonorrhea (1999–2014) were linked using a government-issued, non-duplicated national identification number that is unique and compulsory for each Taiwanese national.

We defined the Taipei metropolitan area as the jurisdiction of Taipei City and New Taipei City, and defined other metropolitan areas as the jurisdiction of cities of Keelung, Taoyuan, Hsinchu, Taichung, Chiayi, Tainan, and Kaohsiung. The other jurisdictions were defined as non-metropolitan areas.

### 1:2 Matched case-control study

We conducted a 1:2 matched case-control study to identify risk factors for AHC among HIV-infected individuals. Each HIV/AHC case was matched to two HIV-infected, non-AHC controls in the NDSS database on age (+/-5 years), age at HIV diagnosis (+/-5 years), sex, mode of transmission (MSM, heterosexual contact, and IDU), date of HIV diagnosis (+/-30 days), and county/city of residence at HIV diagnosis. If a case-patient could not be matched for two controls, the case-patient is excluded from the analysis. If more than two subjects could be identified as controls for a case-patient, we selected the subjects whose age at HIV diagnosis best matched the case-patient. Because IDUs had a high prevalence of chronic HCV infection but the NDSS did not collect chronic HCV data, we excluded IDUs from the case-control study to reduce the possibility of matching HCV-infected controls.

We defined the observation period for a case-patient and controls in each pair as the interval between HIV diagnosis and AHC diagnosis of the case-patient. The end of observation was defined as the case-patient’s date of AHC diagnosis. To ensure a similar observation period in each pair, subjects were not eligible as controls if they died before the date of the corresponding case-patients’ AHC diagnosis. Information on previous reporting of syphilis and gonorrhea, and the last CD4 count, PVL, and ART use by the end of the observation was collected from NDSS. Recent syphilis and recent gonorrhea were defined as any report of syphilis and gonorrhea ≤6 months before the end of observation, respectively.

### Statistical analysis

The trend in proportions was evaluated with the chi-square test for trend. Cases were mapped by using the software Quantum GIS version 1.7.4. We conducted bivariate analyses to compare characteristics of case-patients and controls using the chi-square test or Fisher’s exact test for categorical variables and the Wilcoxon rank sum test for noncategorical variables. All comparisons were two-tailed and a p-value <0.05 was considered significant. Variables that were associated with AHC (p<0.2) in bivariate analyses were considered candidates in a conditional logistic regression model. Adjusted odds ratio (aOR) and 95% confidence intervals were calculated. The analyses were conducted with the statistical software package SAS version 9.4 (SAS Institute, Inc., Cary, North Carolina).

### Ethics statement

Data obtained for the NDSS was for public health surveillance purposes. This study was approved by the Institutional Review Board of TCDC. Informed consent was not obtained because the data used in this study was analyzed anonymously.

## Results

Of 6,624 reported AHC cases to TCDC through the NDSS during June 2001–December 2014, 2,395 (36.2%) had documented HCV seroconversion, of which 93 (3.9%) met the case definitions of HIV/AHC.

### Trend of HIV/AHC

**Fig 1:**
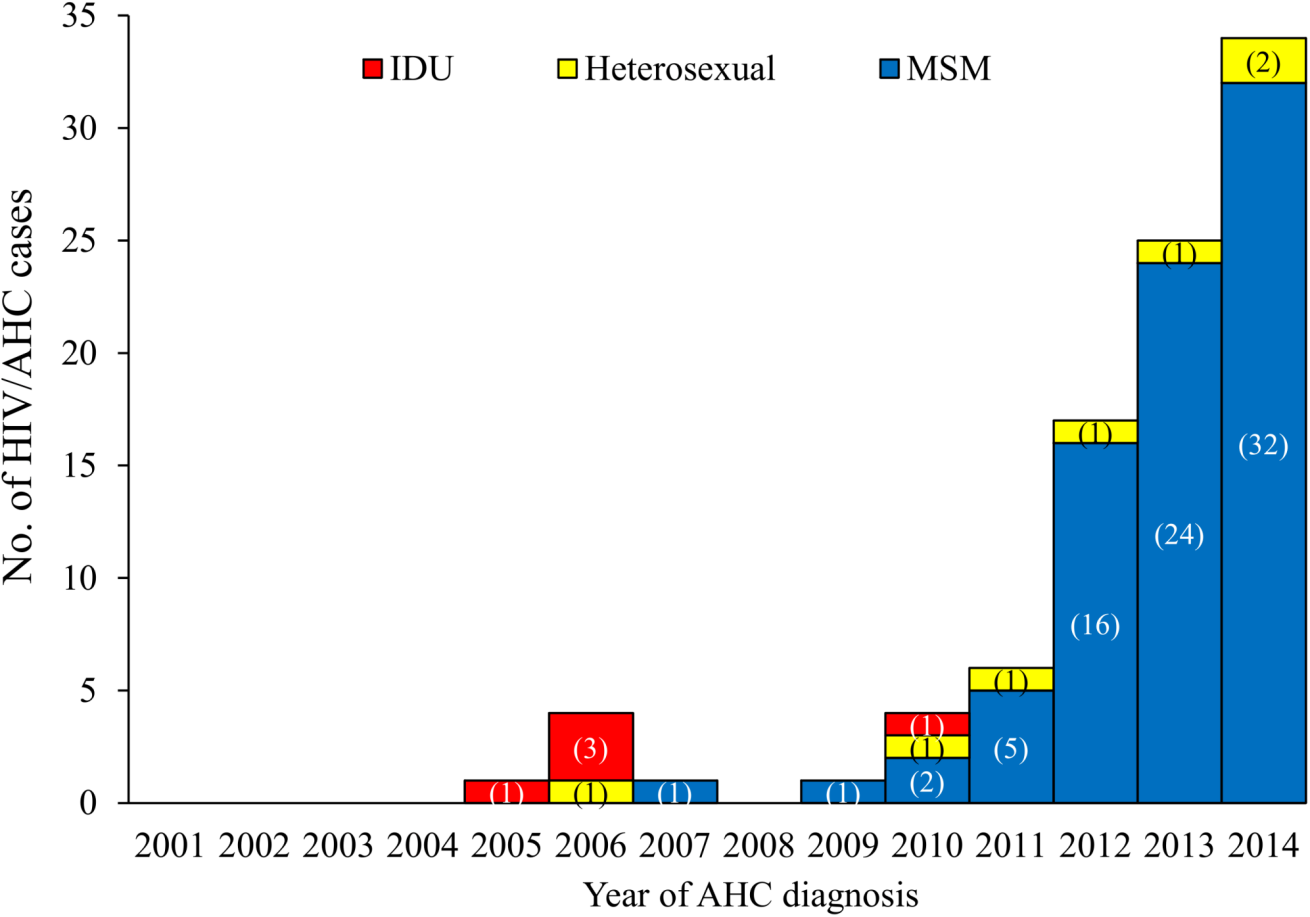
Trend of HIV/AHC cases by population at risk, Taiwan, 2001–2014. ^a^Numbers in parentheses indicate numbers of HIV/AHC cases in each risk category.

The first HIV/AHC case was reported in April 2005, and 5 HIV/AHC cases (three IDUs, one heterosexual, and one MSM) were reported during 2006–2007. The annual number of HIV/AHC cases consistently increased from one case in 2009 to 34 cases in 2014 (Fig 1). Of 87 HIV/AHC cases reported during 2009–2014, MSM, heterosexuals, and IDU accounted for 80 (92%), 6 (6.9%), and one (1.1%) cases, respectively.

**Fig 2:**
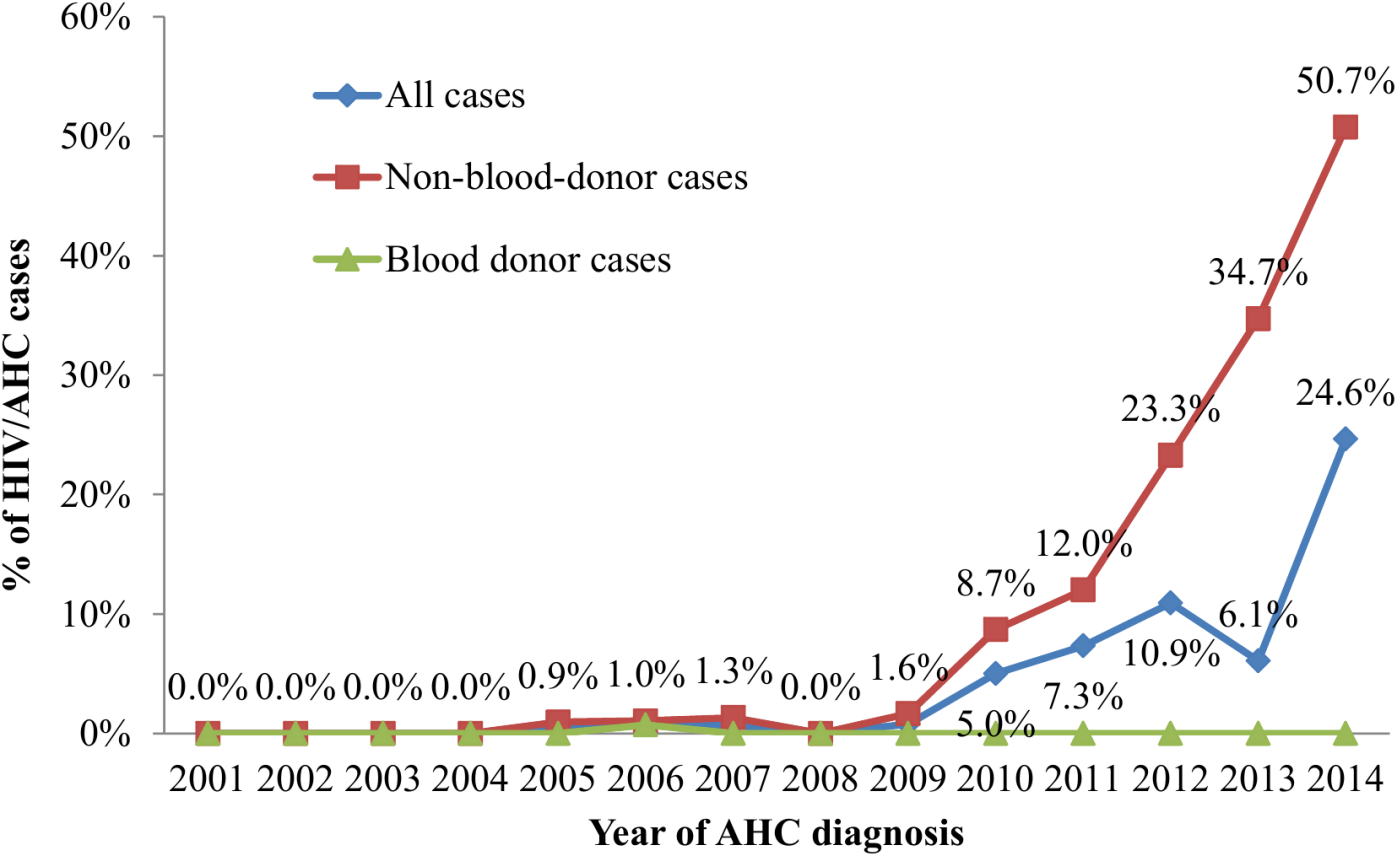
Proportion of HIV/AHC cases among AHC cases with documented HCV seroconversion, Taiwan, 2001–2014.

The proportion of HIV/AHC cases among AHC cases with documented HCV seroconversion reported annually was 0.6−0.8% during 2005–2007 and increased from 0.8% in 2009 to 24.6% in 2014 but was inconsistently lower (6.1%) in 2013 (Fig 2). To remove confounding of excessive reporting from blood donation centers in 2013, we separately analyzed blood-donor cases and non-blood-donor cases. Of 1,445 blood-donor cases with documented HCV seroconversion, three (0.2%) were HIV/AHC cases (two IDUs and one heterosexual), all reported in 2006. Of 950 non-blood-donor cases with documented HCV seroconversion, the proportion of HIV/AHC cases reported annually was overall 0.9%–1.3% during 2005–2007 and consistently increased from 1.6% in 2009 to 50.7% in 2014 (p<0.001) (Fig 2).

### Characteristics of HIV/AHC cases

**Fig 3:**
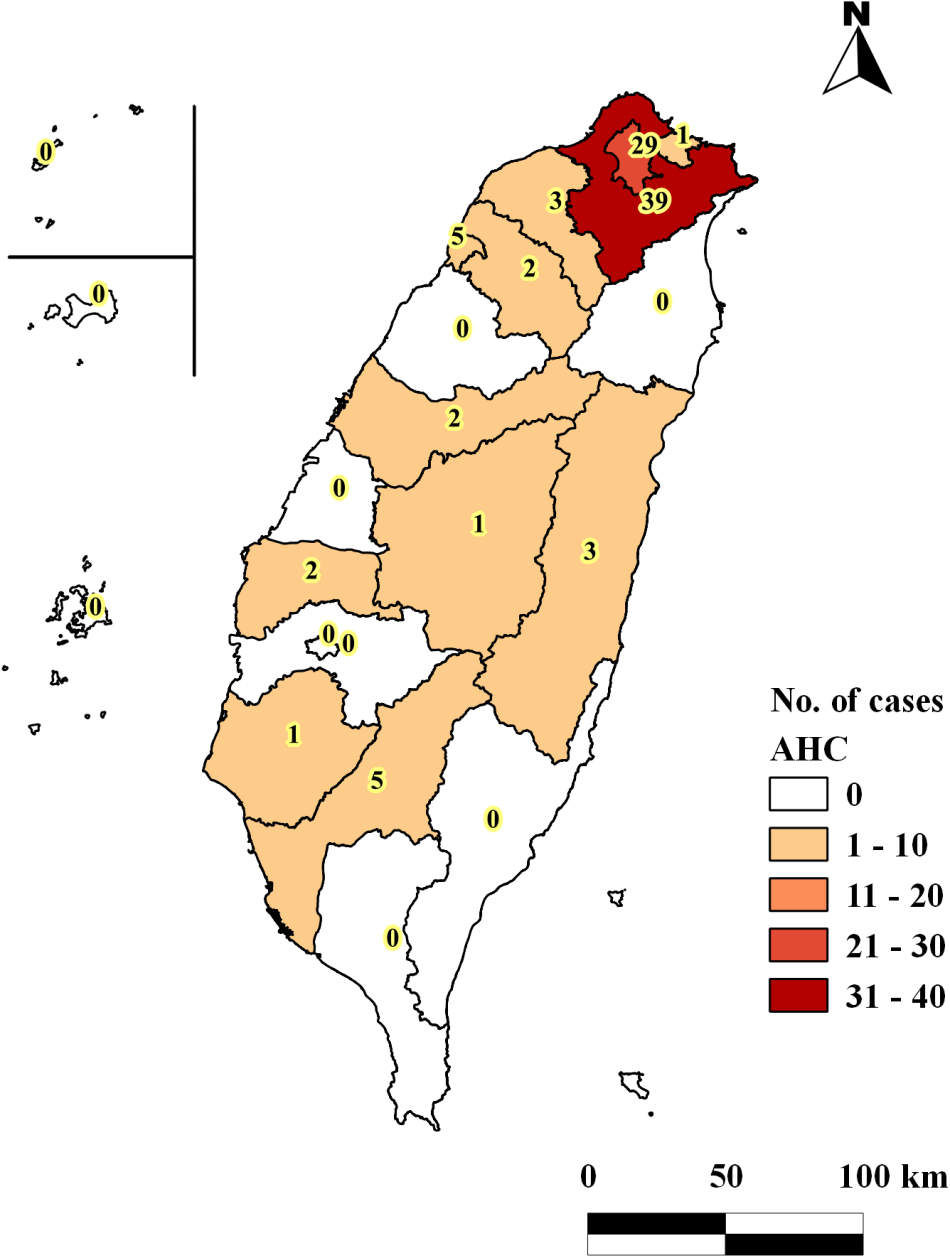
Number of HIV/AHC cases in each county and city, Taiwan, 2001–2014 ^a^Numbers in the map indicate numbers of HIV/AHC cases in each city or county. This map is made by using the basemap provided by Taiwan Ministry of Interior under the Open Government Data License.

Of 93 HIV/AHC cases reported during June 2001−December 2014, all were males with a median age of 34 years (range, 21–49) at the time of AHC diagnosis. HIV diagnosis was made within a median of 1,467 days (range, 2–5,923) before AHC diagnosis. Fig 3 showed geographic distribution of 93 HIV/AHC cases. The majority of HIV/AHC patients were MSM (87%), residents in the Taipei metropolitan areas (73%), and not hospitalized (90%) at the time of AHC diagnosis (Table 1). Approximately half (49%) had symptoms compatible with acute hepatitis, and 82% had an elevated ALT level ≥100 IU/L (median, 206; range, 100–1,808). Syphilis and gonorrhea had been reported in 77% and 20% of the HIV/AHC cases, respectively. Of the HIV/AHC cases with a previous syphilis or gonorrhea report, the median interval from the last reports of syphilis and gonorrhea to AHC diagnosis was 431(range, 0–3,005) and 623 days (range, 104–4,958), respectively.

**Table 1:** Characteristics of HIV-infected individuals with acute hepatitis C—Taiwan, 2001–2014

Characteristics at AHC diagnosis	HIV/AHC
	(N = 93)
	No. (%)
Age group, years	
21–30	36 (39)
31–40	39 (42)
41–50	18 (19)
Male sex	93 (100)
Mode of HIV transmission	
Male–male sex	81 (87)
Heterosexual	7 (8)
Injection drug use	5 (5)
Area of residence	
Taipei metropolitan area	68 (73)
Other metropolitan areas	17 (18)
Non-metropolitan areas	8 (9)
Symptoms compatible with acute hepatitis	46 (49)
Serum aminotransferase level ≥100 IU/L	76 (82)
Hospitalized	9 (10)
Previous syphilis report	72 (77)
Previous gonorrhea report	19 (20)

Abbreviations: AHC, acute hepatitis C; HIV, human immunodeficiency virus

Of 93 HIV/AHC cases, the median CD4 count was 519 cells/mm^3^ (range, 27–1208; 20 [22%] with CD4 count <350 cells/mm^3^) and the median PVL was undetectable (range, <20 to 1.2 x 10^6^ copies/ml; 63 [68%] with a PVL <400 copies/ml) at the time of AHC diagnosis, and 70 (75%) had received ART before AHC diagnosis.

Of 16 HIV/AHC cases with available HCV virologic data, all were MSM with AHC diagnosed during 2012−2014 and HCV viral load were detectable in 15 (94%) with the median HCV viral load of 5.7 x 10^6^ copies/mL (range, 2.8 x 10^3^–1.2 x 10^8^). Two patients were not tested for HCV genotypes because they lost to follow up. HCV genotype data were available in 13 patients. Detected genotypes were 2a (n = 6), 1b (n = 5), 1b + 2a (n = 1) and 2b (n = 1) (Table 2).

**Table 2:** HCV virologic characteristics of 16 HIV-infected individuals with acute hepatitis C at the National Taiwan University Hospital—Taiwan, 2009–2014.

Patient No.	AHC Diagnosis Year	HCV viral load (copies/mL)	HCV genotype
1	2012	3.8 x 10^8^	1b
2	2013	5.7 x 10^6^	Not tested (loss to follow up)
3	2013	6.6 x 10^6^	2b
4	2013	2.8 x 10^3^	2a
5	2013	1.7 x 10^7^	1b
6	2013	1.7 x 10^5^	1b + 2a
7	2013	3.5 x 10^4^	1b
8	2013	1.2 x 10^8^	2a
9	2013	2.7 x 10^4^	2a
10	2014	1.6 x 10^7^	1b
11	2014	7.2 x 10^5^	2a
12	2014	1.5 x 10^5^	1b
13	2014	3.3 x 10^5^	2a
14	2014	1.8 x 10^7^	Not tested (loss to follow up)
15	2014	3.7 x 10^7^	2a
16	2014	Undetectable	NA

Abbreviations: AHC, acute hepatitis C; HIV, human immunodeficiency virus; HCV, hepatitis C virus; NA, not available.

Of 31 HIV/AHC cases reported between March 6 and December 31, 2014, HCV RNA testing results were positive in 14 cases, not conducted in 16 cases, and missing in one case. No HIV/AHC case was reported as HCV RNA negative.

### Sexual and parenteral exposures before AHC diagnosis

Of 31 HIV/AHC patients reported during March 7−December 31, 2014, 28 (90%) completed telephone interviews, two declined, and one was not contactable. Of 28 HIV/AHC patients successfully interviewed, 26 (93%) were self-reported MSM and two were heterosexuals. Of 26 MSM with HIV/AHC, 12 (46%) reported engagement in unprotected sex with a median of 1.5 male partners (range, 1−10) and one reported unprotected sex with a female partner ≤3 months before AHC diagnosis. The two heterosexuals with HIV/AHC denied recent unprotected sex. Nine (32%) of 28 HIV/AHC patients reported parenteral exposures ≤6 months before AHC diagnosis reported in, including intravenous or intramuscular injection for health reasons (n = 4), dental procedures (n = 3), surgery (n = 2), and sharing shavers or toothbrushes (n = 1) (Table 3).

**Table 3:** Sexual and parenteral exposures before diagnosis of acute hepatitis C among HIV-infected individuals—Taiwan, March 7–December 31, 2014.

Exposures associated with HCV acquisition before AHC diagnosis	HIV/AHC (N = 28)
	No. (%)
Unprotected sex ≤3 months	13 (46)
Sex partner of same-sex	12 (42)
No. of sex partners ≥2	6 (21)
Parenteral exposures ≤6 months	9 (32)
Injection for health reason	4 (14)
Dental procedures	3 (11)
Surgery	2 (7)
Shaver/toothbrush sharing	1 (4)
Needle-sharing	0 (0)
Blood component therapy	0 (0)
Hemodialysis	0 (0)
Acupuncture/blood-letting	0 (0)
Occupational needlestick injury	0 (0)
Tattoo/body-piercing	0 (0)

Abbreviations: AHC, acute hepatitis C; HIV, human immunodeficiency virus

### 1:2 Matched case-control study

Of 88 HIV/AHC, non-IDU cases reported during June 2001−December 2014, 79 cases (all MSM) were successfully matched to 158 HIV/non-AHC controls, all of which were reported during 2007−2014. Of 9 unsuccessfully matched cases, we identified only one matched control for 7 cases (5 heterosexuals and 2 MSM) individually and no matched controls for the other 2 heterosexual cases. Table 4 summarized comparisons of characteristics between HIV/AHC cases and matched HIV/non-AHC controls. In bivariate analysis, HIV/AHC cases were significantly associated with a higher last CD4 count and a syphilis report during the observation period (Table 4). In the conditional logistic regression model, factors independently associated with HIV/AHC were having the last syphilis report ≤6 months (recent syphilis) (aOR, 9.9; 95% CI, 3.9–25.2) and having the last syphilis report >6 months (aOR, 2.8; 95% CI, 1.2–6.5) during the observation period (Table 5).

**Table 4:** Characteristics of HIV/AHC cases and matched HIV/non-AHC controls.

Characteristics during observation period	Cases (N = 79)	Controls (N = 158)	*P* Value
Age (years) [median (IQR)]	32 (28−38)	32 (28−38)	0.81
Age at HIV diagnosis (years) [median (IQR)]	28 (24−32)	28 (24−32)	0.78
Year of HIV diagnosis [no. (%)]			
1996−2002	10 (13)	20 (13)	1.00
2003−2008	29 (37)	58 (37)	
2009−2014	40 (51)	80 (51)	
Observation period (months) [median (IQR)]	52 (23−87)	52 (23−88)	1.00
Last CD4 count (cells/mm^3^) [median (IQR)]	525 (209−707)	466 (346−592)	0.02
Last CD4 count (cells/mm^3^) [no. (%)]			
<200	0 (0)	6 (4)	0.15
200−349	16 (20)	37 (23)	
350−499	20 (25)	48 (30)	
≥500	43 (54)	67 (42)	
Last PVL (log_10_ copies/ml) [median (IQR)]	1.3 (1.3−3.5)	1.3 (1.3−3.8)	0.68
Last PVL (log_10_ copies/ml) [no. (%)]			
<400	56 (71)	107 (68)	0.62
≥400	23 (29)	51 (32)	
Antiretroviral-experienced [no. (%)]	63 (80)	115 (73)	0.24
Previous syphilis report [no. (%)]			
Last report ≤6 months (recent syphilis)	31 (39)	16 (10)	<0.001
Last report >6 months	32 (41)	64 (41)	
None	16 (20)	78 (49)	
Previous gonorrhea report [no. (%)]			
Last report ≤6 months	2 (3)	1 (1)	0.09
Last report >6 months	12 (15)	12 (8)	
None	65 (82)	145 (92)	

Abbreviations: AHC, acute hepatitis C; HIV, human immunodeficiency virus; IQR, interquartile range; PVL, plasma viral load

**Table 5:** Characteristics associated with HIV/AHC in conditional logistic regression.

Characteristics during observation period	Adjusted OR (95% CI)	*P* Value
Last CD4 count (per 100 cells/mm^3^ increase)	1.1 (0.98–1.3)	0.08
Previous syphilis report		
Last report ≤6 months (recent syphilis)	9.9 (3.9–25.2)	<0.001
Last report >6 months	2.8 (1.2–6.5)	0.01
None	Reference	Reference
Previous gonorrhea report [no. (%)]		
Last report ≤6 months	1.9 (0.7–5.0)	0.19
Last report >6 months	5.2 (0.4–60.0)	0.19
None	Reference	Reference

Abbreviations: AHC, acute hepatitis C; HIV, human immunodeficiency virus; OR, odds ratio; CI, confidence intervals

To examine if the 9 unsuccessfully matched cases could make a difference to the results, we included all 88 HIV/AHC, non-IDU cases and 165 matched controls in an unconditional logistic regression model. The results still showed that factors independently associated with HIV/AHC were recent syphilis (aOR, 8.9; 95% CI, 3.9–20.3) and having the last syphilis report >6 months (aOR, 2.2; 95% CI, 1.1–4.5) during the observation period.

## Discussion

To our knowledge, this is the first study that describes HIV/AHC epidemiology based on national surveillance data. The findings demonstrate a rapidly increasing trend of HIV/AHC in Taiwan since 2009, characterized by HIV-infected MSM as the major affected population. HIV/AHC was predominantly identified in MSM aged 21–40 years who resided in the Taipei metropolitan area. Risk factors include recent unprotected sex and multiple sex partners, and a past history of syphilis, particularly recent syphilis, is associated with AHC among HIV-infected individuals. These results are consistent with the results of a prior study that first reported increased incidence of recent HCV infection during 2006−2010 in a MSM-predominant HIV cohort in the Taipei metropolitan area [33]. In that study, HCV acquisition within 3 years before enrollment was also associated with recent syphilis (≤6 months) but behavioral risk factors were not explored. Our study applies the European AIDS Treatment Network AHC case definitions, HCV seroconversion within 12 months [38], and the findings confirm that AHC continues as an emerging STD among HIV-infected MSM nationwide and is associated with behavior factors indicating increased sexual risks.

We also demonstrate MSM has predominated over IDU as the major population at risk for HIV/AHC in Taiwan. The findings coincide with a changing epidemiology of HIV infection domestically. In the early 2000s, HCV seroprevalence in Taiwan was 41% for IDUs, 5−9% for female prostitutes, and 4% for male prostitutes [40]. During 2004−2008, a large HIV epidemic was identified among IDUs, characterized by extremely high HCV seroprevalence (>96%) and parenteral exposures including sharing needles, syringes, diluent, and drug paraphernalia [41–44]. Prompt implementation of harm reduction programs that provided IDUs with free clean syringes and methadone led to rapid decline of new HIV cases after 2006 [45]. MSM have become the most preferentially affected population for HIV infection since 2008 [46]. Domestic studies showed HCV seroprevalence (5.5%) and incidence among HIV-infected MSM increased during 2006–2010, whereas HCV seroprevalence remained low (1.1%) among HIV-negative MSM [30, 47]. The alarming upsurge in AHC among HIV-infected MSM suggest that sexual transmission, particularly male−male sex, has recently replaced injection drug use as the major route of HCV transmission among HIV-infected individuals in Taiwan. Recently a 2.2-foldincrease of HCV incidence from 2006−2013 was reported among voluntary counseling and testing clients in Taiwan [48]. Whether HCV infection has increased among sexually active HIV-negative populations warrants further investigation.

The predominant HCV genotypes (2a and 1b) during 2012−2014 identified in the limited sample of this study were identical with the HCV genotypes reported earlier among HIV-infected MSM from the same medical center but different from the predominant HCV genotypes (1a, 6a, and 3a) among IDUs in Taiwan, indicating independent HCV transmission networks in these two populations [30, 41, 48]. Phylogenetic analysis would help determine whether continued or clustered HCV transmission among HIV-infected MSM has occurred across the two study periods. Whereas HCV genotypes 3a and 6a were identified in the previous study, we identified a case infected with HCV genotype 2b, a genotype that was not previously reported among MSM with HIV/AHC in Taiwan but recently reported in Japan associated with non-spontaneous HCV clearance [35]. Surveillance efforts are needed to monitor circulation of HCV genotype 2b and the impact on spontaneous HCV clearance rate among HIV-infected MSM.

The association of AHC with syphilis in our study supports the contributing role of ulcerative STDs to HCV acquisition through impairing mucosal integrity [14, 18, 19, 28, 29, 33, 49]. The increasing HIV/AHC cases and incidence might have been driven by the recently demonstrated rising incidence of syphilis among HIV-infected young Taiwanese men (aged 15−34 years) [50]. A recent study on HIV-infected MSM in Taipei metropolitan areas also concluded association of syphilis with HIV serosorting and recreational drug use, suggesting the facilitating roles of these behavioral factors for STD acquisition [51]. On the other hand, the disproportionate geographic predilection for HIV/AHC in Taipei metropolitan area can be only partially explained by the estimates that 37% of HIV-infected Taiwanese resided in Taipei City and New Taipei City [52]. Regional difference in risky behaviors might play a more important role, as exemplified by an internet-based survey in 2012 that showed MSM in northern Taiwan used recreational drugs and engaged in drug-fueled unprotected anal sex more commonly than MSM in central or southern Taiwan [53]. Notably, although studies in the Western countries have associated HIV/AHC with methamphetamine use among MSM, the 2012 survey indicated that MSM in southern Taiwan used amphetamine more commonly than MSM in northern or central Taiwan [53]. The reasons for such divergence remained unstudied; our anecdotal observation suggested that cheaper cost of domestically produced methamphetamine in southern Taiwan and relative preference of nontraditional imported recreational drugs in northern Taiwan might be contributing factors. Comprehensive investigations of sexual behaviors and recreational drug use among HIV/AHC patients, taking regional differences into account, are recommended to provide guidance for risk assessment and public health interventions against HCV transmission among HIV-infected MSM.

Although the national HIV treatment guidelines have recommended testing of liver function every 6 months and HCV antibody every 12 months for HIV-infected individuals since 2010, adherence to guidelines was inadequate and HCV testing among HIV-infected individuals has been generally triggered by elevated ALT levels [54]. However, ALT-triggered HCV testing strategy might lead to missing 21% of HIV/AHC cases [55]. A recent study using a Monte Carlo computer simulation model demonstrated 6-month ALT and 12-month HCV antibody testing a cost-effective screening strategy for AHC among HIV-infected MSM when the HIV incidence was ≤1.25 cases/100 person-years [56]. Because the NDSS did not collect data on chronic HCV infection, we were not able to have a correct denominator for estimating incidence of AHC among HIV-infected individuals. However, a recent single-center study with service populations in the Taipei metropolitan area estimated that the incidence of recent hepatitis C among HIV-infected MSM was 1.23 per 100 person-years, an incidence level in support of the currently endorsed screening strategy (6-month ALT and 12-month HCV) [30, 56]. Given the regional variation in the number of AHC cases, prospective or retrospective cohort studies that collect baseline and follow-up data on HCV infection to determine the incidence of HIV/AHC nationwide would provide evidence to inform the appropriate screening strategy for AHC among HIV-infected populations at the national and regional levels.

This study is subject to at least the following limitations. First, because our case definitions require documented HCV seroconversion within 12 months, the surveillance data is expected to underestimate AHC disease burden and overrepresent HIV-infected individuals because of enhanced access and frequency of HCV screening among this population. Furthermore, decreasing AHC case detection over the study period is expected because ALT levels and jaundice were added into the clinical criteria of reporting after 2006 and 2010, respectively. Given the limitations, the fact that we still demonstrate an increasing trend of HIV/AHC suggests there is true upsurge of case numbers and the detected cases are only the tip of the iceberg. Second, MSM status and behavioral data were self-reported, collected through public health interviews, and thus subject to socially-desirable biases that could lead to underreported male–male and unprotected sex. Third, our surveillance system depends exclusively on HCV antibody tests before March 6, 2014, not supplemented by RIBA or RNA results in positive AHC cases other than blood donors. Such screening strategy has been reported to have <5%−60% false-positive rates among varied populations [36]. However, the zero HCV RNA negative result among HIV/AHC cases after the NDSS started to collect HCV RNA data in March, 2014 suggested that false-positive results might be rare in our sample. Finally, misclassification might have occurred because we did not have data on chronic HCV infection among the control subjects, leading to underestimation of the crude and adjusted odds ratios for the associated factors.

We recommend health authorities in Taiwan continue surveillance of the trend of HIV/AHC and work with public and private sectors to promote awareness of AHC among HIV-infected men. Healthcare providers should maintain a high index of suspicion of AHC and provide HCV counseling and testing for HIV-infected men with a past history of syphilis, particularly those with recent syphilis.
